# Debridement ability of the WaveOne Gold and TruNatomy systems in the apical third of root canals: *ex vivo* assessment

**DOI:** 10.1590/0103-6440202405773

**Published:** 2024-07-22

**Authors:** Sara Carvalho Avelar de Oliveira, Carlos Eduardo da Silveira Bueno, Rina Andréa Pelegrine, Carlos Eduardo Fontana, Alexandre Sigrist de Martin, Carolina Pessoa Stringheta

**Affiliations:** 1 Faculdade São Leopoldo Mandic, Instituto de Pesquisas São Leopoldo Mandic, Endodontia, Campinas, SP, Brazil; 2 Center for Life Sciences, Postgraduate Program in Health Sciences, Pontifícia Universidade Católica de Campinas, Campinas, SP, Brazil

**Keywords:** dental instruments, debridement, endodontics, root canal preparation, technology

## Abstract

Cleaning and shaping the root canal system are essential steps for performing successful endodontic therapy, and are challenging procedures in the apical region. This study aimed to conduct an *ex vivo* assessment of the debridement ability of the WaveOne Gold (Medium 35/.06) and TruNatomy (Medium 36/.03) systems in the apical third of round root canals of mandibular premolars. Forty-eight teeth, extracted for orthodontic or periodontal reasons, were divided into three groups (n=16), as follows: Group C, control (without instrumentation or irrigation); Group WOG, instrumentation with WaveOne Gold; Group TN, instrumentation with TruNatomy. A total of 40 mL of 2.5% sodium hypochlorite and 5 mL of 17% ethylenediamine tetraacetic acid were used per root canal in all the groups. Ten 0.5-μm serial cross-sections per specimen were obtained every 0.2 mm from a 2-mm segment of the apical region, extending from 1 to 3 mm short of the root apex. The sections were stained with hematoxylin-eosin and analyzed under a digital microscope (100x). The percentages of unprepared walls and remaining debris were quantified using ImageJ software. Generalized linear models were used to analyze the results (α=5%). Groups WOG and TN had significantly lower percentages of unprepared walls and remaining debris than Group C (p<0.05). There was no significant difference between groups WOG and TN for either of the variables studied (p>0.05). Instrumentation with the WaveOne Gold Medium and TruNatomy Medium instruments was associated with equivalent percentages of unprepared walls and remaining debris in the apical third of round canals of mandibular premolars.

## Resumo

A limpeza e modelagem do sistema de canais radiculares são etapas fundamentais para o sucesso da terapia endodôntica e são procedimentos desafiadores na região apical. O objetivo deste trabalho foi realizar uma avaliação *ex vivo* da capacidade de debridamento dos sistemas WaveOne Gold (Medium 35/.06) e TruNatomy (Medium 36/.03) no terço apical de canais radiculares circulares de pré-molares inferiores. Quarenta e oito dentes que haviam sido extraídos por razões ortodônticas ou periodontais foram divididos em três grupos (n=16),da seguinte forma: Grupo C, Controle (sem instrumentação nem irrigação); Grupo WOG, instrumentação com WaveOne Gold; Grupo TN, instrumentação com TruNatomy. Um total de 40 mL de hipoclorito de sódio a 2,5% e 5 mL de ácido etilenodiamino tetraacético a 17% foi utilizado por canal radicular em todos os grupos. Dez secções transversais em série de 0,5 μm por espécime foram obtidas a cada 0,2 mm de um segmento de 2 mm da região apical, estendendo-se desde 1 até 3 mm aquém do ápice radicular. As seções foram coradas com hematoxilina-eosina e analisadas sob um microscópio digital (100x). As porcentagens de paredes não tocadas e detritos remanescentes foram quantificadas utilizando-se o *software* ImageJ. Modelos lineares generalizados foram utilizados para analisar os resultados (α=5%). Os grupos WOG e TN apresentaram porcentagens significativamente menores de paredes intocadas e detritos remanescentes do que o grupo C (p<0,05). Não houve diferença significativa entre os grupos WOG e TN quanto a nenhuma das variáveis estudadas (p>0,05). A instrumentação com os instrumentos WaveOne Gold Medium e TruNatomy Medium foi associada a porcentagens equivalentes de paredes intocadas e detritos remanescentes no terço apical de canais circulares de pré-molares inferiores.

## Introduction

Cleaning and shaping of the root canal system (RCS) are essential to performing successful endodontic treatment. In this respect, the instruments used must be concomitantly safe and effective in promoting disinfection and facilitating the filling of the RCS ^1^. Mechanized instrumentation systems optimize the root canal shaping process; however, evidence shows that mechanized instruments fail to touch every wall of the RCS, regardless of the technique applied ^2^. The apical region is considered the most critical for debridement and disinfection since the apical foramen and ramifications are closely connected to periradicular tissues. Furthermore, uninstrumented areas may harbor remnants of pulp tissue and bacterial biofilm ^3^ ^,^ ^4^.

Apical diameter enlargement significantly influences the degree of cleanliness and disinfection of the root canal, thus increasing the chances of successful endodontic treatment. An increased apical enlargement enables a higher percentage of canal walls to be instrumented ^5^, thereby enhancing the removal of infected dentin and biofilm. Additionally, it promotes improved irrigant flow within the apical third of the canal, hence leading to a more substantial reduction in bacterial load ^6^. Accordingly, Pérez et al. ^7^ demonstrated a significant increase in the percentage of prepared canal wall surfaces, according to each increase in instrument size during apical preparation. These findings seem to indicate that the main reason why some areas of the main canal remain unprepared is that the final instrument used to prepare them is smaller than the largest diameter of the original canal. The main concern associated with the use of instruments that produce smaller apical diameters is whether or not they can ensure adequate cleaning of the root canal space. Minimal canal enlargement has been associated with inadequate removal of debris, smear layer, pathogenic bacteria, infected dentin, and pulp remnants ^8^. However, there is growing concern about the potential for excessive enlargement of the root canal, owing to the risk of its causing unnecessary removal of tooth structure, hence rendering the tooth more susceptible to fracture ^9^.

Instrumentation performed in rotary motion has already proven capable of reducing operative time and effectively shaping the RCS, because the nickel-titanium (NiTi) rotary instruments promote a more centralized preparation than manual files, and preserve the original anatomy of the root canals more effectively ^1^ ^,^ ^10^. However, continuous rotation can produce considerable cyclic and/or torsional fatigue of the instruments inside the root canals, thus increasing the risk of fracture. Accordingly, reciprocating rotation was introduced as an alternative to continuous rotation, since its kinematics reduces instrument stress, and allows preparation of the entire RCS with a single instrument ^11^. Despite the different kinematics of continuous rotation and reciprocating instruments, studies have demonstrated that they have similar shaping capabilities ^2^ ^,^ ^12^. Instrument cross-section also plays an important role in its resistance to fatigue, in that its geometric characteristics may have an impact on the amount of contact area between the instrument and the root canal wall ^13^ ^,^ ^14^. Advances in metallic alloys have prompted the development of instruments with increased flexibility and cyclic fatigue resistance, and the heat treatments applied to the instruments have allowed controlling their shape. As a result, access to difficult-to-reach areas of the RCS has been facilitated, ledge formation during instrumentation is avoided, and preparations that better preserve the original anatomy of the canal can be obtained ^15^.

The instruments of the WaveOne Gold system (Dentsply Sirona, Ballaigues, Switzerland) undergo a thermal treatment that induces the formation of a Ti_3_Ni_4_ layer on their surface. This leads to a predominantly martensitic alloy, resulting in increased elasticity of the metal. The system consists of a glide path creation instrument (Glider, 15/.02), and four shaping instruments (20/.07, 25/.07, 35/.06, and 45/.05) that have regressive taper up to the end of their working part, and a parallelogram-shaped cross-section, with one or two cutting edges ^16^.

The TruNatomy system (Dentsply Sirona) was developed following the current trend toward more conservative preparations and improved preservation of pericervical dentin ^17^. Its instruments undergo a heat treatment that imparts greater flexibility and less shape memory to the NiTi alloy, compared to conventional and M-Wire alloys. This allows precurving the instrument to facilitate access to the canal orifice. The system comprises a cervical preparation file (Orifice Modifier, 20/.08) that has a fixed taper and a 7-mm working tip, a glide path creation file (Glider, 17/.02) that has a regressive taper and a centered parallelogram-shaped cross-section, and three shaping files (20/.04, 26/.04, and 36/.03) that have regressive taper and an off-centered parallelogram-shaped cross-section ^18^.

To date, and to the best of our knowledge, there are no studies in the literature comparing the WaveOne Gold and TruNatomy systems concerning their root canal debridement effectiveness in terms of unprepared walls and debris remaining after instrumentation. Histological analysis performed with a digital microscope is considered a well-established method for this purpose (19-21). Thus, this study aimed to conduct an *ex vivo* evaluation of the debridement ability of the WaveOne Gold (Medium, 35/.06) and TruNatomy (Medium, 36/.03) mechanized instrumentation systems in the apical third of circular canals of mandibular premolars. The null hypothesis was that there would be no significant differences between the tested instrumentation systems regarding the variables studied.

## Materials and methods

This study was approved by the local research ethics committee (Approval n. 5.358.820). The 48 mandibular premolars used in the study were donated expressly by patients whose teeth were indicated for extraction for orthodontic or periodontal reasons. All the donating patients signed a free and informed consent form. The pulp vitality of the teeth was confirmed before extraction using a cold spray test (Endo-Frost; Roeko, Langenau, Germany). Only teeth displaying a normal response to the test were collected. After extraction, the teeth were stored in a biorepository and kept in a bottle containing 10 mL of 10% buffered formalin until the beginning of the study ^22^. The total period used for tooth collection was 3 months ^20^ ^,^ ^21^.

### Sample size

Considering a test power of 0.80, a significance level of 5%, and a large effect size (f = 0.47; ^23^ ^,^ ^24^), the sample size required for the study was calculated at 16 specimens per group. This sample size is consistent with that of a previous study ^20^. G*Power 3 software v. 3.1.9.2 (Heinrich-Heine-Universität Düsseldorf, Düsseldorf, Germany) ^25^ was used to perform the sample size calculation.

### Specimen selection

The inclusion criteria were fully formed roots, root curvatures of up to 20°, circular canals-i.e. buccolingual diameter less than twice the mesiodistal diameter at 3 mm short of the radiographic apex of the canal, as confirmed on digital radiographs in the buccolingual and mesiodistal directions ^19^-and initial diameter of the apical foramen corresponding to a #15 K-type file (Dentsply Sirona). Teeth with calcifications, root dilacerations, pathological root resorptions (internal, external, or apical), perforations (internal or external), or root caries, visible under a stereomicroscope (EK3ST; Eikonal Equipamentos Ópticos e Analíticos, São Paulo, SP, Brazil) at 8x magnification or previous endodontic treatment were excluded.

### Specimen preparation

All the teeth were accessed endodontically with high-speed diamond burs. A #15 K-type file (Dentsply Sirona) was introduced into the canal until its tip was visible exiting the apical foramen under an operating microscope (Decius, DF Vasconcelos, Rio de Janeiro, RJ, Brazil) at 20x magnification. The working length (WL) was established at 1 mm short of the apical foramen ^2^. The root surfaces were scraped with periodontal curettes (Golgran, São Caetano do Sul, SP, Brazil), and the specimens were disinfected in a 0.1% thymol solution for 24 h. After this period, the specimens were kept in distilled water until the beginning of the root canal instrumentation stage ^2^.

### Root canal instrumentation

Initially, the apical foramen of each root was covered with a light-cured gingival barrier (Top Dam; FGM, Joinville, SC, Brazil) to create a closed environment for the chemical-mechanical preparation ^19^. The 48 specimens were divided into two experimental groups and one control group (n = 16). The three groups were paired for mesiodistal diameter (mm), buccolingual diameter (mm), and angle of apical curvature (°). The Kruskal-Wallis test was used to confirm the group pairing. The R program (R Foundation for Statistical Computing, Vienna, Austria. https://www.R-project.org/) was used to perform the calculations, and a significance level of 5% was used.

In Group C (control), the root canals were neither instrumented nor irrigated. In Group WOG (WaveOne Gold), the WOG Glider instrument (15/.02) was used initially to pre-enlarge the root canal using in-and-out movements, until reaching the WL. Subsequently, the WOG Medium instrument (35/.06) was used in a sequence of three in-and-out movements with an average amplitude of 3 mm, in the cervical, middle, and apical thirds, until reaching the WL. The reciprocating kinematics and the torque and speed settings used were those pre-programmed by the manufacturer for the WOG system in the endodontic motor used (X-Smart IQ; Dentsply Sirona).

In Group TN (TruNatomy), the Orifice Modifier (20/.08) was used initially to pre-enlarge the root canal using in-and-out movements restricted to the cervical and middle thirds. Then, the TN Glider instrument (17/.02) was used in a sequence of three in-and-out movements, until reaching the WL. Subsequently, the TN Medium instrument (36/.03) was used in a sequence of three in-and-out movements with an average amplitude of 3 mm, in the cervical, middle, and apical thirds, until reaching the WL. Instrumentation was performed using the same motor used in the previous group, but in continuous rotation, and with speed and torque settings of 500 rpm and 1.5 N.cm, respectively, following the manufacturer's recommendations for the TN system.

**Figure 1:**
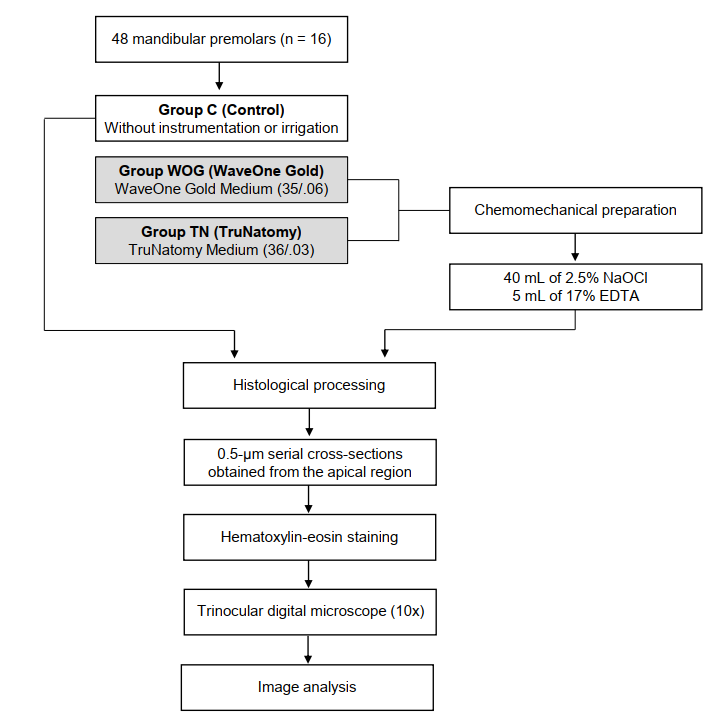
Flowchart of the experimental procedures performed in the study.

All the specimens were instrumented by the same operator, who was experienced in the systems tested. Each instrument was used to prepare four canals in both experimental groups and then discarded ^2^. Apical patency was checked at each instrument change with a #15 K-type file (Dentsply Sirona). Irrigation with 2.5% sodium hypochlorite (NaOCl) was performed at each instrument change, or every three in-and-out movements, using a 5 mL hypodermic syringe and a 31-gauge NaviTip needle (Ultradent, South Jordan, UT, USA). Following the chemomechanical preparation, the specimens were irrigated with 5 mL of 17% ethylenediamine tetraacetic acid for 1 min and then received a final irrigation with 5 mL of 2.5% NaOCl. The total volume of NaOCl solution used per canal was 40 mL. The irrigant was aspirated with a capillary tip (Ultradent), the canals were dried with absorbent paper tips (Dentsply Sirona), and the specimens were prepared for histological analysis (Figure 1).

### Histological analysis

After the specimens were removed from the device made for the cleaning and shaping steps, the canals were passively filled with 10% buffered formalin using a 10-mL disposable hypodermic syringe and a 31-gauge needle (Ultradent). Subsequently, the specimens were immersed in 10% buffered formalin and then stored for 24 h in the same solution for fixation, until the beginning of the histological processing stage. The specimens were decalcified in a 20% formic acid solution (Merck, São Paulo, SP, Brazil) for 15 days, and washed under running water for 8 h. They were then dehydrated in solutions with decreasing concentrations of ethanol, cleared in xylol, and embedded in histologic paraffin (Synth, Diadema, SP, Brazil) using a tissue processor (Leica TP 1020; Leica Biosystems, Nussloch, Germany). Next, 0.5-μm thick serial cross-sections were cut with a microtome (Leica RM 2245; Leica Biosystems, São Paulo, SP, Brazil) every 0.2 mm of a 2-mm segment extending from 1 to 3 mm short of the root apex, for a total of 10 sections per specimen. The sections were cut with a disposable razor (Duraedge, Fremont, OH, USA), then spread over a glass slide (Prolab, São Paulo, SP, Brazil), stained with hematoxylin-eosin, and photographed with a digital camera (Tucsen Prime HD, Tucsen Photonics, Fujian, China) coupled to a trinocular digital microscope (Eclipse 100-LED, Nikon Instruments, Kawasaki, Japan) under 100x magnification ^21^. The images were transferred to a workstation and saved in JPG format.

The percentage of uninstrumented root canal walls was determined by calculating the perimeter of the canal left unprepared by the instruments about the total perimeter of the canal, using ImageJ software (National Institute of Health, Bethesda, MD, USA, https://imagej.nih.gov/ij/). The criterion used to identify unprepared walls was the presence of surface irregularities, characterized by an abrupt change in canal wall continuity and partial removal of pre-dentin ^21^. The percentage of remaining debris (dentin chips, residual necrotic pulp, and particles loosely attached to the canal wall) was determined using the same software, by dividing the area occupied by the debris observed in each specimen by the total area of the canal lumen. All 960 analyses (3 groups x 16 specimens x 10 sections x 2 variables) were performed by a single-blinded and calibrated examiner ^21^.

### Statistical analysis

The percentage of unprepared walls considered for each specimen was the average of the percentages observed in the 10 histological sections analyzed. Descriptive and exploratory analyses of the data were performed, and a generalized linear model was applied. An analysis considering the three study groups was conducted, and, since no variation was observed in the control group, a second analysis was performed excluding this group.

The mean percentage of remaining debris considered for each specimen was the average of the percentages observed in the 10 histological sections analyzed. Since the descriptive and exploratory analyses indicated that the data failed to meet the assumptions of an analysis of variance (ANOVA), a generalized linear model was applied to assess the effect of the group on the percentage of remaining debris. All the analyses were performed using the R program (R Foundation for Statistical Computing), and the level of significance used was 5%.

## Results

**Figure 2:**
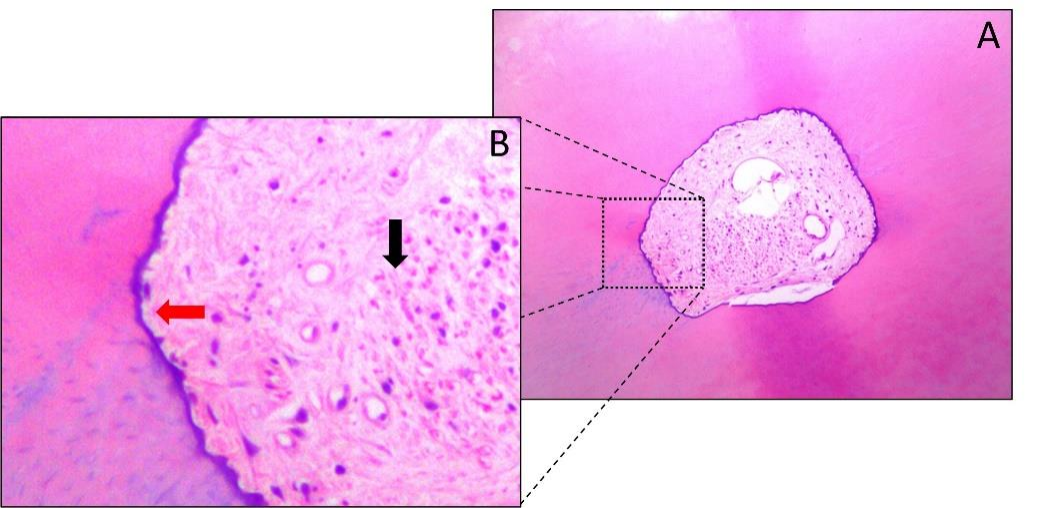
Representative photomicrographs of the cross sections of the apical region of mandibular premolars from the control group (without instrumentation or irrigation). (A) 100x magnification. (B) 400x magnification; note the presence of organic and inorganic tissue (black arrow) and intact walls (red arrow).

Figures 2 through 4 show representative images of the histological sections obtained from the apical segment for the three study groups (extending from 1 to 3 mm short of the apex). Table 1 shows that groups WOG and TN had significantly lower percentages of unprepared walls than the control group (p < 0.05), but indicates that there was no significant difference between the two experimental groups with respect to this variable (p > 0.05). Since no variability was observed in the control group (standard deviation = 0.0%), an analysis was also performed considering only the two experimental groups, and this second analysis confirmed that there was no significant difference between them (p > 0.05).

**Figure 3:**
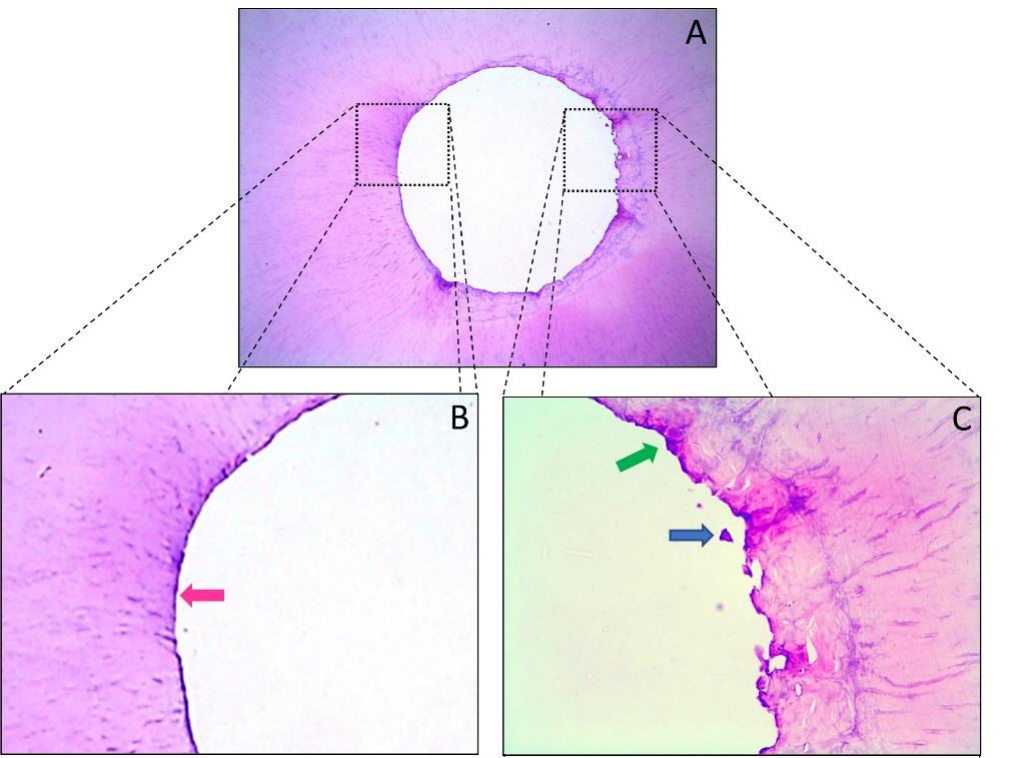
Representative photomicrographs of the cross sections of the apical region of mandibular premolars from Group WOG (WaveOne Gold Medium, 35/.06). (A) 100x magnification. (B) 400x magnification; note the presence of instrumented walls, with regular contours and no debris (pink arrow). (C) 400x magnification; note the area with unprepared walls (green arrow) and with the occasional presence of remaining debris (blue arrow).

**Figure 4:**
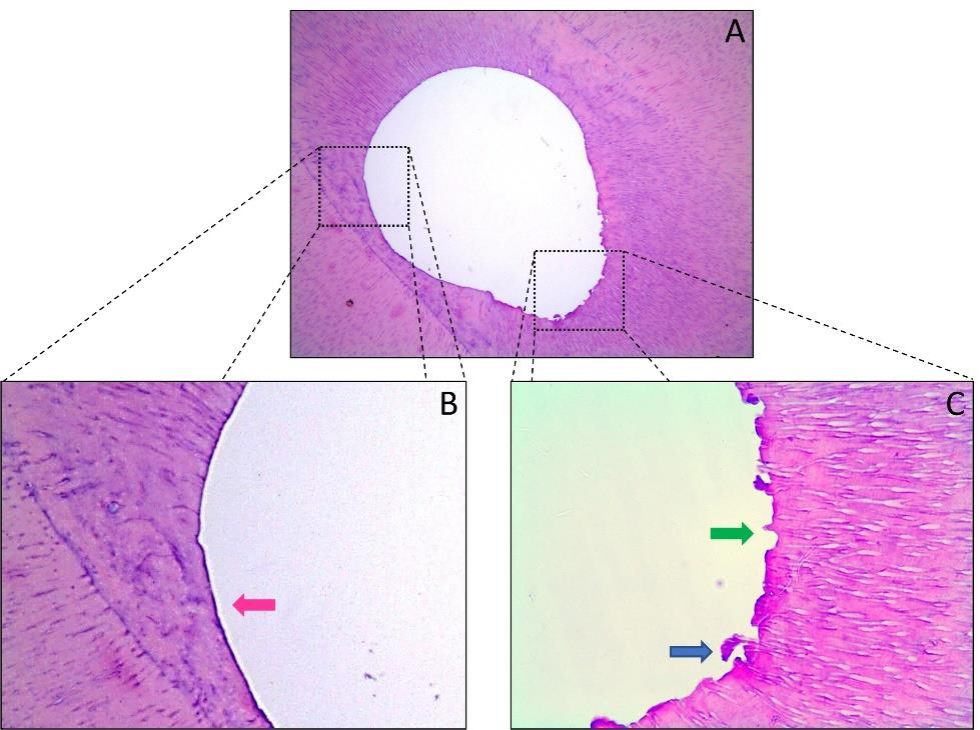
Representative photomicrographs of the cross sections of the apical region of mandibular premolars from Group TN (TruNatomy Medium, 36/.03). (A) 100x magnification. (B) 400x magnification; note the presence of instrumented walls, with regular contours and no debris (pink arrow). (C) 400x magnification; note the area with unprepared walls (green arrow) and with the occasional presence of remaining debris (blue arrow).

**Table 1:** Percentage of untouched walls according to study group.

Group	Mean (standard deviation)	p-value	p-value
Control	100.00% (0.0%) ^a^	< 0.0001^*^	0.6808^**^
WaveOne Gold	57.02% (18.10%) ^b^		
TruNatomy	54.73% (13.70%) ^b^		

Different lowercase letters indicate a statistically significant difference (p ≤ 0.05); ^*^: p-value considering the three study groups; ^**^: p-value considering only the experimental groups (TruNatomy and WaveOne Gold).

Table 2 shows that the control group had a significantly higher percentage of remaining debris than groups WOG and TN (p < 0.05), but indicates that there was no significant difference between the two experimental groups with respect to this variable (p > 0.05).

**Table 2:** Percentage of remaining debris according to study group.

Group	Mean (standard deviation)	p-value
Control	37.51% (21.66%) ^a^	< 0.0001
WaveOne Gold	0.82% (1.56%) ^b^	
TruNatomy	0.48% (0.92%) ^b^	

Different lower-case letters indicate a statistically significant difference (p ≤ 0.05).

## Discussion

No significant difference was found between groups WOG and TN with respect to the two variables studied. Therefore, the null hypotheses were not rejected. The present study evaluated instruments (sizes 35/.06 and 36/.03) that produce apical preparations with similar diameters, to minimize the risk of methodological bias, and allow comparison of the parameters associated with the two systems tested. There is no consensus in the literature regarding the ideal apical diameter to ensure effective cleaning and disinfection of the RCS, and, as a result, a favorable prognosis in terms of periapical tissue repair. However, wider apical preparations promote greater intracanal bacterial reduction, and provide more favorable results when treating teeth with apical periodontitis ^7^ ^,^ ^26^. Lima et al. ^27^ demonstrated that the use of a reduced taper system (Bassi Logic, .03) during root canal preparation resulted in a greater percentage of unprepared canal areas when compared to XP-endo Shaper and Reciproc instruments. These results indicate that a larger apical diameter will likely include a greater number of anatomical variations, touch a greater percentage of canal walls, and promote a more abundant flow of irrigants. Furthermore, more effective chemical and mechanical action will likely be attained, because a wider apical diameter allows the irrigation needle to be inserted up to a point comparatively closer to the apical foramen, as demonstrated in the studies conducted by Boutsioukis, Arias-Moliz ^28^ and de Oliveira et al. ^29^.

Lee et al. ^20^ and Stringheta et al. ^21^ pointed out that irrigant agitation during and/or at the end of instrumentation can have a positive effect on canal debridement. Lee et al. ^20^ demonstrated that ultrasonic activation of the irrigant in small apical preparations resulted in root canals that were as clean as preparations with larger diameters. This finding was common to both round and oval canals. Similarly, Stringheta et al. ^21^ evaluated the influence of apical preparation size and final irrigation protocol-whether conventional syringe, ultrasonically activated irrigation, or XP-endo Finisher-on the debridement of the apical third of oval canals in mandibular molars and concluded that both ultrasonic activation and XP-endo Finisher were associated with lower percentages of remaining debris than conventional irrigation. The absence of an irrigant agitation protocol may be considered a limitation. However, the present study aimed to evaluate the debridement ability of the instruments tested *per se*. This is why only conventional syringe irrigation was included, thus ruling out the potential influence of this additional variable on the research results.

An instrumentation finalized with instruments of smaller apical diameter, although in line with the current trend of conservative preparations, may result in a smaller reduction of pathogens, and greater amounts of contaminated dentin and remnants of pulp tissue, thus increasing the chances of reinfection after endodontic treatment. In this regard, instruments should ideally be able to perform an adequate apical preparation, but without promoting excessive wear in the cervical region. This way, the structure of healthy dentin would be preserved, and fracture resistance would be ensured after placement of a coronal restoration, while concomitantly ensuring adequate cleaning of the most apical portion of the root canal and adequate debridement of its walls ^17^ ^,^ ^30^. Instruments of the TN system are manufactured from a metallic wire with an initial diameter of 0.8 mm, unlike conventional mechanized instruments, manufactured with a wire whose initial diameter is 1.1 mm ^18^. It's important to note that this instrument aims to enhance the preservation of pericervical dentin, even when performing wider apical preparations, e.g., when using the system’s Medium file (36/.03).

Silva et al. ^31^ compared the TN and ProTaper Gold systems with respect to their ability to preserve pericervical dentin and enlarge the apical portion of mandibular molar root canals. Their results demonstrated that the TN system produced lower levels of both cervical wear in the mesial roots and apical transportation in the mesiobuccal canals. The authors concluded, however, that the amount of dentin removed by both systems tested was so small that these differences could be considered clinically insignificant. Vorster et al. ^32^ compared the TN and WOG systems in the canals of mandibular molars and demonstrated that the TN Glider and TN Prime instruments promoted significantly faster preparations than the WOG Glider and WOG Primary instruments, using both traditional and conservative approaches. The authors associated these results with the regressive taper and thinner design of the TN instruments, which ensured their effectiveness even in more conservative access cavities.

The present study used mandibular premolars with similar anatomy to minimize the potential bias associated with morphological variations. Overly wide canals could compromise the study’s ability to assess the desired parameters, which is why premolars with circular canals and initial apical diameter compatible with a #15 type K-file were selected. Although the internal anatomy of these premolars favors instrument contact with the canal walls, the observed rates of unprepared walls, associated with both instrumentation systems tested herein, were above 50%. The rates of unprepared root canal surfaces reported in the literature vary significantly, which may be related to anatomical differences among the different types of teeth used ^33^. The results found in the present study are compatible with those found by Lee et al. ^20^, who compared apical preparations with different diameters, regarding the degree of cleansing associated with them. Even though these authors used only rotary instrumentation with the EdgeFile X7 system, they used a similar histological analysis methodology to assess the percentage of unprepared walls in circular canals of mandibular premolars. Pérez Morales et al. ^34^ compared the shaping ability of six different mechanized instrumentation systems, including TN and WOG, using computed microtomography (micro-CT), and concluded that the WOG instruments were able to touch a greater percentage of canal walls, albeit producing greater changes to the original root canal anatomy. In contrast, the TN instruments promoted greater preservation of the original root canal anatomy but touched a smaller percentage of their walls. However, it should be noted that these results refer to the entire extension of the evaluated canals, unlike the results of the present study, which refer only to their apical portion.

The two types of kinematics evaluated in the present study-continuous rotation and reciprocating rotation-had no significant influence on the rates of unprepared walls or remaining debris, thus corroborating the results found by Stringheta et al. ^2^, who evaluated four mechanized instrumentation systems using micro-CT-two continuous and two reciprocating rotation-and who found similar rates of unprepared surfaces and remaining debris. Guedes et al. ^12^ also compared two continuous rotation and two reciprocating systems regarding canal volume, canal area, canal-to-root width ratio, and unprepared surface areas, as well as regarding their ability to promote a centered preparation. The authors found that all systems had similar performance among all of the variables evaluated. In contrast, the literature reviews published by Ahn et al. ^35^ and Sousa-Neto et al. ^36^ concluded that reciprocating instrumentation was associated with greater apical extrusion of debris, and explained that this observation could be associated with greater accumulation of debris in the apical region, since the level of escape of material in the cervical direction associated with this type of instrumentation is smaller. The present study found percentages of remaining debris close to zero in both experimental groups. This may be related to both the circular anatomy of the canals-favoring irrigant flow and instrument contact with canal walls-and the total volume of 40 mL of NaOCl used per canal. This result is compatible with those found by Lacerda et al. ^19^ and Pérez et al. ^37^, who performed histological analyses and observed several specimens that were entirely free of debris in the apical portion at the end of the chemical-mechanical preparation. Perez et al. ^37^ also compared rotational and reciprocating kinematics, but did so using the XP-endo Shaper and Reciproc Blue systems. They pointed out that abundant irrigation with NaOCl is a determining factor in eliminating biofilm and residual debris when instrumentation has been finalized.

The validity of the histological approach used in the present study was confirmed by the presence of pulp tissue in the canals of specimens from the control group. Although tissue processing procedures fail to fully preserve all the properties inherent to vital pulp tissue, this approach has been widely used in the literature to investigate the debridement of canal walls at a histological level. The processed pulp tissue undergoes a certain degree of shrinkage and can detach from the canal walls, which is why the proportion of the root canals filled by pulp tissue in the control group was not 100% ^20^ ^,^ ^21^. Another limitation of the histological method is that it can provide images only in two dimensions. In this regard, the small thickness required for the cross-sections used in this method may not reflect the reality of the entire volume of the assessed canal. Nevertheless, this limitation can be partially circumvented by evaluating multiple serial sections, whose individual assessments can then be combined to provide a more reliable picture of the entire specimen, as in the present study, which analyzed ten sections per specimen, exclusively from the apical region. Furthermore, although group homogeneity was statistically confirmed concerning both apical curvature angle and buccolingual and mesiodistal diameters (circular canals), a radiograph examination performed to ascertain the root canal shape is not the most accurate experimental methodology. Therefore, further investigation using micro-CT is warranted to ensure greater accuracy during the stage of specimen standardization.

Micro-CT is currently the gold standard for assessing mineralized tissues since it allows identifying and quantifying data related to mechanized instrumentation of the RCS, its internal anatomy, and changes to the original trajectory of the canals after treatment, including apical transportation, zipping, and perforations ^38^ ^,^ ^4^. De-Deus et al. ^39^ demonstrated the effectiveness of this method also to assessing pulp tissue by applying a tissue impregnation protocol that included a potassium triiodide solution known as Lugol's solution. This solution promotes a radiocontrast effect on pulp tissue so that it can be identified on micro-CT images. Further investigation is still required to validate this new methodology; however, because of its non-destructive nature, it is already clear that this technique allows assessment of both the individual and combined effects of the chemical dissolution and mechanical removal of pulp tissue promoted by cleaning and shaping procedures.

The clinical significance of the present study is that its results seem to indicate that root canal instrumentation performed with instruments that provide a more conservative preparation, with less wear of the tooth structure, can promote a level of apical debridement similar to that promoted by instrumentation with conventional instruments, irrespective of the kinematics applied. Future research is also needed to assess complementary irrigation methods for optimizing cleaning, decontamination, and removal of residual debris, and concomitantly reducing the removal of mineralized tissue during endodontic preparation to a minimum, to extend the longevity of the involved tooth in function.

It was concluded that instrumentation with the WOG (Medium file) and TN (Medium file) systems was associated with equivalent percentages of unprepared walls and debris remaining in the apical third of circular canals of mandibular premolars.

## References

[1] Hülsmann M Peters OA, Dummer PMH (2005). Mechanical preparation of root canals: shaping goals, techniques and means. Endod Topics.

[2] Stringheta CP, Bueno CES, Kato AS, Freire LG, Iglecias EF, Santos M (2019). Micro-computed tomographic evaluation of the shaping ability of four instrumentation systems in curved root canals. Int Endod J.

[3] Plotino G, Özyürek T, Grande NM, Gündoğar M (2019). Influence of size and taper of basic root canal preparation on root canal cleanliness: a scanning electron microscopy study. Int Endod J.

[4] Siqueira JF, Pérez AR, Marceliano-Alves MF, Provenzano JC, Silva SG, Pires FR (2018). What happens to unprepared root canal walls: a correlative analysis using micro-computed tomography and histology/scanning electron microscopy. Int Endod J.

[5] Aazzouzi-Raiss K, Ramírez-Muñoz A, Mendez S PM, Vieira GCS, Aranguren J, Pérez AR (2023). Effects of conservative access and apical enlargement on shaping and dentin preservation with traditional and modern instruments: a micro-computed tomographic study. J Endod.

[6] Sabeti MA, Saqib Ihsan M, Aminoshariae A (2024). The effect of master apical preparation size on healing outcomes in endodontic treatment: a systematic review and meta-analysis. J Endod.

[7] Pérez AR, Alves FRF, Marceliano-Alves MF, Provenzano JC, Gonçalves LS, Neves AA (2018). Effects of increased apical enlargement on the amount of unprepared areas and coronal dentine removal: a micro-computed tomography study. Int Endod J.

[8] Fatima S, Kumar A, Andrabi SMUN, Mishra SK, Tewari RK (2021). Effect of apical third enlargement to different preparation sizes and tapers on postoperative pain and outcome of primary endodontic treatment: a prospective randomized clinical trial. J Endod.

[9] Usta SN, Silva EJNL, Falakaloğlu S, Gündoğar M (2023). Does minimally invasive canal preparation provide higher fracture resistance of endodontically treated teeth? A systematic review of in vitro studies. Restor Dent Endod.

[10] Ferreira F, Adeodato C, Barbosa I, Aboud L, Scelza P, Zaccaro Scelza M (2017). Movement kinematics and cyclic fatigue of NiTi rotary instruments: a systematic review. Int Endod J.

[11] Pedullà E, Grande NM, Plotino G, Gambarini G, Rapisarda E (2013). Influence of continuous or reciprocating motion on cyclic fatigue resistance of 4 different nickel-titanium rotary instruments. J Endod.

[12] Guedes IG, Rodrigues RCV, Marceliano-Alves MF, Alves FRF, Rôças IN, Siqueira JF (2022). Shaping ability of new reciprocating or rotary instruments with two cross-sectional designs: an ex vivo study. Int Endod J.

[13] Roda-Casanova V, Pérez-González A, Zubizarreta-Macho A, Faus-Matoses V (2022). Influence of cross-section and pitch on the mechanical response of NiTi endodontic files under bending and torsional conditions - a finite element analysis. J Clin Med.

[14] Kim E, Ha JH, Dorn SO, Shen Y, Kim HC, Kwak SW (2023). Effect of heat treatment on mechanical properties of nickel-titanium instruments. J Endod.

[15] Gavini G, Santos MD, Caldeira CL, Machado MEL, Freire LG, Iglecias EF (2018). Nickel-titanium instruments in endodontics: a concise review of the state of the art. Braz Oral Res.

[16] Martins JNR, Silva EJNL, Marques D, Belladonna F, Simões-Carvalho M, Vieira VTL (2021). Design, metallurgical features, mechanical performance and canal preparation of six reciprocating instruments. Int Endod J.

[17] Clark D, Khademi J (2010). Modern molar endodontic access and directed dentin conservation. Dent Clin North Am.

[18] Van der Vyver PJ, Vorster M, Peters OA (2019). Minimally invasive endodontics using a new single-file rotary system. Int Dent - Afr Ed.

[19] Lacerda MFLS, Marceliano-Alves MF, Pérez AR, Provenzano JC, Neves MAS, Pires FR (2017). Cleaning and shaping oval canals with 3 instrumentation systems: a correlative micro-computed tomographic and histologic study. J Endod.

[20] Lee OYS, Khan K, Li KY, Shetty H, Abiad RS, Cheung GSP (2019). Influence of apical preparation size and irrigation technique on root canal debridement: a histological analysis of round and oval root canals. Int Endod J.

[21] Stringheta CP, Pelegrine RA, Montalli VAM, Gutmann JL, Bueno CES (2021). Influence of apical preparation size and final irrigation protocol on the debridement of oval root canals. Braz Dent J.

[22] De-Deus G, Barino B, Zamolyi RQ, Souza E, Fonseca A, Fidel S (2010). Suboptimal debridement quality produced by the single-file F2 ProTaper technique in oval-shaped canals. J Endod.

[23] Cohen J (1988). Statistical power analysis for the behavioral sciences.

[24] Cohen J (1992). A power primer. Psychol Bull.

[25] Faul F, Erdfelder E, Lang AG, Buchner A (2007). G*Power 3: a flexible statistical power analysis program for the social, behavioral, and biomedical sciences. Behav Res Methods.

[26] Rodrigues RCV, Zandi H, Kristoffersen AK, Enersen M, Mdala I, Ørstavik D (2017). Influence of the apical preparation size and the irrigant type on bacterial reduction in root canal-treated teeth with apical periodontitis. J Endod.

[27] Lima CO, Barbosa AFA, Ferreira CM, Augusto CM, Sassone LM, Lopes RT (2020). The impact of minimally invasive root canal preparation strategies on the ability to shape root canals of mandibular molars. Int Endod J.

[28] Boutsioukis C, Arias-Moliz MT (2022). Present status and future directions - irrigants and irrigation methods. Int Endod J.

[29] de Oliveira RA, Weissheimer T, Só GB, da Rosa RA, Souza MA, Ribeiro RG (2022). Dentinal tubule penetration of sodium hypochlorite in root canals with and without mechanical preparation and different irrigant activation methods. Restor Dent Endod.

[30] Augusto CM, Barbosa AFA, Guimarães CC, Lima CO, Ferreira CM, Sassone LM (2020). A laboratory study of the impact of ultraconservative access cavities and minimal root canal tapers on the ability to shape canals in extracted mandibular molars and their fracture resistance. Int Endod J.

[31] Silva EJNL, Lima CO, Barbosa AFA, Lopes RT, Sassone LM, Versiani MA (2022). The impact of TruNatomy and ProTaper Gold instruments on the preservation of the periradicular dentin and on the enlargement of the apical canal of mandibular molars. J Endod.

[32] Vorster M, van der Vyver PJ, Markou G (2022). The effect of different molar access cavity designs on root canal shaping times using rotation and reciprocation instruments in mandibular first molars. J Endod.

[33] Pacheco-Yanes J, Gazzaneo I, Campello AF, Marceliano-Alves MF, Estrela C, Bueno MR (2022). Planned apical preparation using cone-beam computed tomographic measures: a micro-computed tomographic proof of concept in human cadavers. J Endod.

[34] Pérez Morales MLN, González Sánchez JA, Olivieri JG, Elmsmari F, Salmon P, Jaramillo DE (2021). Micro-computed tomographic assessment and comparative study of the shaping ability of 6 nickel-titanium files: an in vitro study. J Endod.

[35] Ahn SY, Kim HC, Kim E (2016). Kinematic effects of nickel-titanium instruments with reciprocating or continuous rotation motion: a systematic review of in vitro studies. J Endod.

[36] Sousa-Neto MD, Silva-Sousa YC, Mazzi-Chaves JF, Carvalho KKT, Barbosa AFS, Versiani MA (2018). Root canal preparation using micro-computed tomography analysis: a literature review. Braz Oral Res.

[37] Pérez AR, Ricucci D, Vieira GCS, Provenzano JC, Alves FRF, Marceliano-Alves MF (2020). Cleaning, shaping, and disinfecting abilities of 2 instrument systems as evaluated by a correlative micro-computed tomographic and histobacteriologic approach. J Endod.

[38] Peters OA, Laib A, Göhring TN, Barbakow F (2001). Changes in root canal geometry after preparation assessed by high-resolution computed tomography. J Endod.

[39] De-Deus G, Belladonna FG, Cavalcante DM, Simões-Carvalho M, Silva EJNL, Carvalhal JCA (2021). Contrast-enhanced micro-CT to assess dental pulp tissue debridement in root canals of extracted teeth: a series of cascading experiments towards method validation. Int Endod J.

